# Making smart investment decisions in clinical research

**DOI:** 10.1186/s13063-015-1123-1

**Published:** 2015-12-29

**Authors:** Nick Bansback, Edward Keystone, James O’Dell, Ciaran S. Phibbs, Keri Hannagan, Mary Brophy, Aslam Anis

**Affiliations:** School of Population and Public Health, University of British Columbia, Vancouver, BC Canada; Centre for Health Evaluation and Outcome Sciences, St Paul’s Hospital, Vancouver, BC Canada; Arthritis Research Centre for Canada, Vancouver, Canada; University of Toronto, Toronto, Canada; The Rebecca MacDonald Centre for Arthritis & Autoimmune Diseases, Mount Sinai Hospital, Toronto, Canada; Nebraska–Western Iowa Health Care System, Omaha, USA; University of Nebraska Medical Center, Omaha, USA; Health Economics Resource Center and the Center for Health Care Evaluation, Veterans Affairs Palo Alto Health Care System, Menlo Park, CA USA; Department of Health Research and Policy and the Center for Primary Care Outcomes Research, Stanford University School of Medicine, Stanford, CA USA; VA Cooperative Studies Program Coordinating Center, Boston, USA; School of Medicine, Boston University, Boston, USA

**Keywords:** Rheumatoid arthritis, Randomized trials, Economics

## Abstract

A recent trial in rheumatoid arthritis found an inexpensive, but infrequently used, combination of therapies is neither inferior nor less safe than an expensive biologic drug. If the trial had been conducted over 10 years ago, arguably 100’s of millions of dollars since spent on biologics could have been released to other, more effective treatments. Given the ever increasing number of trials proposed, this commentary uses the trial as an example to challenge payers and research funders to make smarter investments in clinical research to save potential future costs.

Trial registration: NCT00405275, registered 29 November 2006

## Background

A recent randomized controlled trial in rheumatoid arthritis (RA) patients determined that a strategy of first adding two Disease-modifying Antirheumatic Drugs (DMARDs) to methotrexate (a combination known as Triple Therapy) is neither inferior nor less safe than first adding the biologic anti-TNF drug etanercept to methotrexate in patients with active disease despite the methotrexate [1]. The Rheumatoid Arthritis Comparison of Active Therapies (RACAT) trial, for which ethical approval was obtained from the institutional review boards for each of the sites in the United States and Canada, strengthens findings of earlier strategy trials that have found triple therapy to be noninferior to biologic agents, [2, 3] and a metaanalysis suggesting no difference in their ability to prevent disease progression [4, 5].

The implication of the study, for which all 353 participants provided written informed consent, is that inexpensive triple therapy - a combination promoted for over a decade but infrequently used - should be initiated prior to commencing expensive biologic therapy in patients whose disease is not completely controlled by methotrexate alone. Furthermore, when patients fail a regimen containing a biologic, they should switch to Triple Therapy if it has not previously been tried instead of presuming that a different biologic regimen is needed. The results are particularly significant from a healthcare cost and sustainability perspective since spending on biologic therapies (which all appear to have near equal efficacy [6]) for patients with RA ranks among the top expenditure category in the formulary budgets of most Western healthcare systems [7]. If implemented in practice, Triple Therapy would delay the use of biologic treatment by at least 6 months without impairing a patient’s disease progression, thereby saving $100’s of millions each year.

It will, however, be a major challenge to change rheumatologists prescribing patterns given their familiarity with biologics and guidelines advocating biologic use [8]. Arguably, the multimillion-dollar question raised by the RACAT trial is why was it not conducted earlier? Both biologics and Triple Therapy were independently shown to be effective in comparison to methotrexate more than 15 years ago, [9, 10] but only recently have been compared (Table 1).

Clinical trials are expensive to conduct, and more trials are proposed than can be funded. Despite this, it seems that priority is rarely given to trials where the ultimate objective is to reduce future costs. While “present and future resource implications” [11] are often considerations in funding research, it is rarely recognized that there exists an opportunity cost associated with each funding decision and that reducing costs has a potential to significantly improve health, by releasing money to be spent on alternative healthcare services that provide greater health benefits. It seems the potential opportunity for a blockbuster that industry recognizes can make enormous profits is not reciprocated by payers and research funders with the opportunity to save an enormous amount of money.

With the benefit of hindsight, the $13 million spent on the RACAT trial would have been recouped many times over if conducted in the early 2000s. By looking back, we consider the evidence and the uncertainties relating to this trial in the early 2000s and reflect if we had the foresight then to fund this trial. Most importantly, we consider the lessons to determine which smart investments should be made in research today to save potential future costs.

## Main text

### Identification

Timing is crucial in investing. By the time an investment is obvious, much of the potential benefit is lost. In the case of trials, they can only be funded if they have been conceived with a plausible hypothesis and clear justification. However, the plausibility of Triple Therapy being equivalent to biologics was established by the early 2000s. A trial published in 1996 demonstrated that Triple Therapy was superior in comparison to MTX alone [7], and this was corroborated in 2002 [12]. While numerous RCTs have been conducted on biologics in RA over the past 15 years [6], regulatory approval has meant nearly all have compared a biologic to placebo or MTX in combination with placebo, with the vast majority in patients showing an inadequate response only to MTX, not Triple Therapy. Table 1 describes how more than 50 trials compared a biologic to MTX before a comparison with Triple Therapy was made.

### Estimated future market size

If biologics had not gone on to become expensive blockbuster drugs, the question of why the RACAT trial was not conducted earlier would be moot. Nevertheless, this could have been predicted; the prevalence of RA is approximately 1 % of the population, of whom it could be predicted at least 10 % would be severe enough to be marketable for biologic therapy. The price of biologics was established with the first drugs of their kind - etanercept and infliximab - and experience tells us that these prices remain stable or indeed increase even when new drugs enter the market. While the actual diffusion of biologics into the market can be difficult to predict, if the pharmaceutical industry could anticipate the potential market for biologics in the early 2000s (numerous pre-clinical trials of other biologics were in existence by 2002 [13]), then there is no reason research funders could not also make these predictions.

### Consideration of risk and reward

It is impossible to fund all trials with all plausible comparators, so the risk a trial will not meet its primary endpoint has to be considered when deciding which research to fund. Even in trials that meet their primary endpoint, limitations exist in all studies, and these limitations can impact whether the results influence clinical practice. It is not clear how funders evaluate these risks, or value what is an acceptable risk. However, simple decision analysis can help discern the expected value of information a trial would provide which can be compared against the potential impact of the trial [14]. In the case of RACAT, the trial would have effectively paid for itself in the first year in Canada alone, so it would have been prudent to fund even if the risk of failure was very high.

Many payers have restricted access to biologics for RA patients that have trialed specified conventional DMARDs. For example, in Canada, reimbursement for biologics by a government payer is only available through a special authority where rheumatologists usually must demonstrate that a number of DMARD combinations have been previously tried. Including Triple Therapy in this list would have been simple and would have increased utilization substantially on the basis of the evidence from a trial like RACAT to provide evidence.

## Conclusions

Whether the RACAT trial will lead to reductions in the use of biologics in patients with RA, and therefore paying for the investment remains to be seen. It is clear that over 10 years ago, the RACAT trial would have been a smart investment – we demonstrate that the anticipated rewards would have indicated the risk was worth taking many times over (Table 2). The result of the trial would have had an influence on jurisdictions beyond the funding agency; however, the case for multinational studies such as RACAT, where the costs are shared and the external validity is strengthened, are even more compelling. With many new expensive treatments are emerging for diseases such as asthma, hepatitis and cancer, among others, a change in thinking about research investments has the potential to save billions of dollars in the next 10 years.

**Table 1:** Clinical trials comparing biologics, Triple Therapy and methotrexate

Year	Biologic vs. MTX	Triple Therapy vs. MTX	Triple Therapy vs. biologic
1996		TRI > MTX	
1997			
1998	INF > MTX		
1999	INF > MTX, ETN > MTX	TRI > MTX, TRI > MTX	
2000	ETN > MTX, INF > MTX, INF > MTX		
2001			
2002	ANA > MTX	TRI > MTX	
2003	ABA > MTX, ADA > MTX		
2004	ANA > MTX, RIX > MTX, ADA > MTX, ETN > MTX, ETN > MTX, INF > MTX		
2005	ABA > MTX, INF > MTX		
2006	INF > MTX, ADA > MTX, RIT > MTX, RIT > MTX, ABA > MTX, TOC > MTX, INF > MTX, INF > MTX		
2007	INF > MTX, ADA > MTX, ABA > MTX		
2008	ADA > MTX, TOC > MTX, ETN > MTX, GOL > MTX, CER > MTX, INF > MTX, TOC > MTX		
2009	ADA > MTX, ADA > MTX, GOL > MTX, TOC > MTX, CER > MTX, ABA > MTX		
2010	RIT > MTX, TOC > MTX, ETN > MTX, GOL > MTX		
2011	TOC > MTX		
2012	CER > MTX, GOL > MTX		TRI ≈ ETN, TRI ≈ INF
2013	ABA > MTX, ADA > MTX, ADA > MTX, ETN > MTX,		TRI ≈ ETN

ABA, Abatacept; ADA, Adalimumab; ANA, Anakinra; CER, Certolizumab; ETN, Etanercept; GOL, Golimumab; INF, Infliximab; RIT, Rituximab; TOC, Tocilizumab; TRI, Triple Therapy (methotrexate, sulfasalazine, hydroxychloroquine)

> indicates superiority in primary endpoint whereas ≈ indicates noninferiority

**Table 2:** Proposed approach for making smarter investments

Stage	Proposal	Illustrative example of rheumatoid arthritis (RA) in early 2000s in Canada
Identification (of the problem)	Scan of pharmaceutical trials in phase 1, 2a/2b to determine which products pharmaceutical companies believe will be a good investment.	Numerous trials of biologic agents in RA from multiple pharmaceuticals would have been identified, suggesting a belief in the potential for biologics to become blockbuster drugs.
Identification (of a potential solution)	Call for studies of alternative, cheaper treatments in clinical contexts identified above.	Triple therapy would have been proposed given the O’Dell trial in 1996.
Estimated market size	Consider the potential market size and assume the price of first to market product to estimate the potential budget impact.	A crude estimate of 0.1 % of the population using the original price of the first biologics (~$18,000 per year) would have led to a prediction of an enormous potential market. In Canada, this would be $500 million per year, or $5 billion, considering 10 years of use.
Consideration of risk and reward	Estimate the cost of the trial – and compare with the expected cost of a successful trial result (probability of trial meeting the primary outcome and subsequently impacting uptake multiplied by the potential). If the cost of trial is greater, then do not fund, but if it is less, then fund.	For a $10 million trial cost, it would only need to have a minimum 0.2 % ($10 million < 0.2 x $5 billion) chance of success for the trial to be deemed a good investment in Canada. Given the evidence at the time, even the most pessimistic assessment would have provided a probability of success larger than 1 %. Hence, using this rationale, the trial would have been funded.

An important issue to consider is who should pay for these clinical trials? Regulatory bodies typically require efficacy studies instead of the comparative effectiveness studies like RACAT, and there is little incentive for pharmaceutical manufacturers to bear the cost. There is a strong argument for regulatory studies to require comparative effectiveness studies, but they tend to be funded through national health research budgets [15]. However, it would have been the payers that would have ultimately saved their budget through this trial. We believe payers need to have a stronger voice in terms of both what regulators require in terms of comparators from phase III studies, and in terms of the way research funders invest in evidence. If the healthcare system is serious about providing quality, cost-effective care, tremendous opportunities remain through smart investments in clinical research.

## Abbreviations

Anti-TNF: anti-tumor necrosis factor
DMARDs: disease-modifying antirheumatic drugs
MTX: methotrexate
RA: rheumatoid arthritis
RACAT: rheumatoid arthritis comparison of active therapies
